# Laryngeal Mask Airway for neonatal resuscitation in a developing country: evaluation of an educational intervention. Neonatal LMA: an educational intervention in DRC

**DOI:** 10.1186/1472-6963-10-254

**Published:** 2010-08-31

**Authors:** Vincenzo Zanardo, Alphonse Simbi, Massimo Micaglio, Francesco Cavallin, Leon Tshilolo, Daniele Trevisanuto

**Affiliations:** 1 Department of Pediatrics, Padua University, (3, Via Giustiniani) Padua (35128), Italy; 2 Department of Pharmacology and Anesthesiology, Padua University, (3, Via Giustiniani) Padua (35128), Italy

## Abstract

**Background:**

Studies carried out in developing countries have indicated that training courses in newborn resuscitation are efficacious in teaching local birth attendants how to properly utilize simple resuscitation devices. The aim of this study was to assess the knowledge and expertise gained by physicians and midwifes who participated in a Neonatal Resuscitation Course and workshop organized in a Third World Country on the use of Laryngeal Mask Airway (LMA).

**Methods:**

A 28-item questionnaire, derived from the standard test contained in the American Heart Association and the American Academy of Pediatrics Neonatal Resuscitation Manual, was administered to 21 physicians and 7 midwifes before and after a course, which included a practical, hands-on workshop focusing on LMA positioning and bag-ventilation in a neonatal manikin.

**Results:**

The knowledge gained by the physicians was superior to that demonstrated by the midwifes. The physicians, in fact, demonstrated a significant improvement with respect to their pre-course knowledge. Both the physicians and the midwives showed a good level of expertise in manipulating the manipulating the manikin during the practical trial session. The midwifes and physicians almost unanimously manifested a high degree of approval of neonatal resuscitation by LMA, as they defined it a sustainable and cost-effective method requiring minimal expertise.

**Conclusions:**

Further studies are warranted to test the advantages and limits of the neonatal LMA training courses in developing countries.

## Background

Intrapartum hypoxia and birth asphyxia are widely regarded as major causes of morbidity and mortality in developing countries [1,2]. All those involved in delivery room care must, consequently, possess the knowledge and expertise to perform neonatal resuscitation [3].

Maintaining a patent airway and providing effective positive pressure ventilation (PPV), which is currently achieved in the delivery room by means of a face mask (FM) or a tracheal tube (TT), is standard treatment and forms the cornerstone of emergency neonatal care in First World countries [2], but making these interventions feasible in settings where resources are limited is particularly challenging [4,5]. Since its introduction into clinical practice, the LMA has gained increasing popularity for resuscitation of adult as well as pediatric patients, and more recently in neonatal resuscitation. Some organizations have, in fact, made it a part of their guidelines and LMA has been included in the AAP and AHA Guidelines of Neonatal Resuscitation Program (NRP) since 2000 [3,6]. It has been seen that LMA provides a low pressure airtight seal against the glottis [7-10] and studies on the efficacy of ventilation by medical and paramedical personnel in neonatal training models have shown that it is characterized by ease of insertion and rapid, adequate airway patency [11]. Although this device cannot be considered a substitute for the TT, it could, at least theoretically, play an ancillary role in developing countries [12] where it offers practical, cost-effective, sustainable advantages over the face mask.

Some studies carried out in Third World countries have indicated that local birth attendants who have been trained in newborn resuscitation are capable of learning and properly utilizing simple resuscitation techniques (e.g. Mouth-to-mask breathing and room air) [4], reducing asphyxia-related deaths [13-16]. It is uncertain, however, what aspects of these training programs were responsible for that outcome.

The aim of this study is to assess the knowledge gained by local birth attendants (physicians and midwifes) who participated in a NRP course and workshop on LMA organized in the Democratic Republic of Congo (DRC).

## Methods

A 3-day NRP course was held by the Continuing Education for Africa (CEFA), in Kinshasa, the DRC, in September 2006. The course consisted of a number of didactic sessions proportionally divided into the seven steps of the NRP: (I) principles of resuscitation; (II) initial steps in resuscitation; (III) bag mask ventilation; (IV) chest compressions; (V) tracheal intubation; (VI) medications; and VII) special considerations [3], followed by practical hands-on training session, including LMA positioning and bag-ventilation in a neonatal manikin (Neonate Airway Trainer; Laerdal, Norway).

Twenty-eight local birth attendants, (21 physicians and 7 midwifes) from the Congo Brazeville (1), Benin (1), Cameroun (1), and the remaining from other areas of the DRC, participated in the course. All of the participants took - both before and after the course - a 28 question test, an adaptation of a standard one contained in the AHA/AAP Neonatal Resuscitation Manual [3] and underwent a practical trial evaluating their proficiency in manipulating LMA and a neonatal manikin. The participants had 60 minutes to complete the test, which included multiple choice, fill-in-the blank, and true/false questions. The test, which was written in French, was strictly supervised.

The questions concerned: (I) features of LMA; (II) advantages of LMA over the face mask; (III) advantages of LMA over the endotracheal tube; (IV) disadvantages of LMA; and (V) potential applications in neonatal resuscitation. Additional file 1

The theoretical test was followed by a practical trial during which the trainees were asked to attempt LMA insertion in a manikin (designed for skills training in neonatal resuscitation) in less than 15 seconds (successful insertion was defined as effective thorax expansion verified by the instructor). The participants were also asked to anonymously express their opinion about their degree of satisfaction (high/low) with the course and the sustainability and cost-effectiveness (yes/no) of LMA in their respective countries.

The study was approved by the Ethical and Scientific Committee of the CEFA Agency.

### Statistical analysis

"Improvement", defined as the difference (number or percentage) between the pre and post test scores, in the two groups (physicians and midwifes) was analyzed separately by the McNemar test and Fisher's exact test, as appropriate. A post hoc multiple comparison analysis using the Bonferroni's adjustment was performed when statistically significant (p < 0.05) differences were found in the groups. A p value <0.05 was considered significant.

## Results

Theoretical knowledge about and practical skills in neonatal resuscitation using LMA on a neonatal manikin gained by the participants (physicians and midwifes) at a neonatal resuscitation course are outlined in Tables 1 and 2.

**Table 1:** Knowledge gained by participants during a NRP training course on neonatal resuscitation focusing on LMA.

Neonatal Laryngeal Mask Airway	Pre-test correct answers n.(%)		Post-test correct answers n.(%)	
**Features**	**Physicians:** n. 21	**Midwifes: n.7**	**Physicians: n.21**	**Midwifes: n.7**

Range	5	3	7	3
Size	6	1	10	3
Lubrication	3	2	11^	4
Tip flattening	5	3	11^	3
Advancing	5	2	10	4
Location	4	1	12^	3
Cuff inflation	5	0	11^	3
Connection for PPV	13	5	11	5
Correct positioning	1	0	14*	7
				
	47/189 (24.8)	17/63 (26,9)	97/189 (51.3)	35/63 (55.5)

**Advantages over the face mask**				

Less skill required	4	0	11^	6
Easy placement	3	1	4	2
Better airtight seal	1	1	12*	3
Improved SO_2 _	3	1	11	6
Function unaffected by anatomical factors	1	1	6	1
Less hand fatigue	7	0	10	7
				
	19/126 (15.0)	4/42 (9.5)	54/126 (42.8)	25/42 (9.52)

**Advantages over the tracheal tube**				

Easier and quicker placement by trained medical and non-medical personnel	5	2	10	4
Avoids laryngoscopy	2	5	14*	7
Avoids tracheal oedema	4	0	14*	6
Efficacy in upper airway malformations when intubation and mask ventilation fail	9	4	14	6
Avoids use of neuromuscular blocking agents	4	3	15*	7
				
	24/105 (22.8)	14/35 (40.0)	67/105 (63.8)	30/35 (85.7)

**Disadvantages**				

Gastric insufflation and aspiration	0	0	8	4
Inadequate alveolar ventilation	13	3	14	6
Impossibility of suctioning the airway	4	3	8	6
Impossibility of administering drug endotracheally	12	3	14	7
				
	29/84 (34.5)	9/28 (32.1)	44/84 (52.3)	17/28 (60.7)

**Potential applications in neonatal resuscitation**				

When face mask and tracheal tube resuscitation fall	10	6	15	7
In neonatal training models allows a patent airway in a shorter time than endotracheal tube	4	0	14*	6
Incidence of failure low with LMA	10	5	15	7
Ruinously	5	2	15*	7
				
	29/84 (34.5)	13/28 (46.4)	59/84 (70.2)	27/28 (96.4)

Pre- and post-test correct answers.

^McNemar test p value (p < 0.05), corrected by Bonferroni's adjustment (p < 0.05)*

**Table 2:** "Improvement", defined as the difference between the pre- and post-test scores.

Neonatal LMA		Improvement n.	**McNemar Test**^**§**^	Improvement *%	Fisher test*
					

**Features**	Physicians	50	p < 0.0001	35.2	p < 0.72
		
	Midwifes	18	p < 0.0001	40.9	

					

**Advantages over the face mask**	Physicians	35	p < 0.0001	32.7	p < 0.02
		
	Midwifes	21	p < 0.0001	55.3	

					

**Advantages over the tracheal tube**	Physicians	43	p < 0.0001	53.1	p < 0.08
		
	Midwifes	16	p < 0.0001	76.2	

					

**Disadvantages**	Physicians	15	p < 0.0001	27.3	p < 0.26
		
	Midwifes	8	p = 0.08	42.1	

					

**Potential applications**	Physicians	30	p < 0.0001	54.5	p < 0.006
		
	Midwifes	14	p = 0.001	93.3	

Facial mask, FM; tracheal tube, TT

^§ ^Improvement (n) = a pre-test wrong answer coupled with a post-test correct answer by McNemar's test. *Improvement (%) = improvement number over pre-test wrong answer number ratio by Fischer's test. p value (p < 0.05).

Compared with the initial score, the overall knowledge gained by the physicians and midwifes who participated in the NRP course and workshop on neonatal resuscitation by LMA was increased, but with different percentages regarding the single test questions. In particular, physicians significantly improved in 12/28 post-test items analyzed by McNemar's test and in 7/12 by Bonferroni's adjustment. Table 1

The "improvement", defined by the number of correct answers in post-test coupled with a wrong answer in pre-test, was statistically significant for the physicians and midwifes with reference to the questions concerning their theoretical knowledge about LMA. The "improvement", defined by the percent of correct answers in post-test coupled with a wrong answer in pre-test, with regard to the questions dealing with the advantages of LMA over the facial mask (p < 0.02) and its potential applications (p < 0.005) was statistically significant for the physicians alone. Table 2

The degree of approval by the physicians and midwifes of neonatal resuscitation by LMA and their opinions regarding its sustainability and cost-effectiveness are outlined in Table 3.

**Table 3:** Practical trial in inserting LMA in a neonatal manikin.

LMA::	Physicians: n. 21	Midwifes: n. 7
**Successful placement:**		

- number of attempts:		
1	20	6
2	0	1
3	1	0

		

**Insertion time**:		

- positioning < 15 seconds	20	6
- positioning > 15 seconds	1	1

		

**Degree of approval:**		

- high	20	7
- low	1	0

**Sustainability:**		

- yes	20	7
- no	1	0

**Cost-effectiveness:**		

- yes	20	7
- no	1	0

The practical part of the test showed that the physicians and midwifes were skillful in their attempts at LMA insertion. One physician and one midwife failed, respectively, 3 and 2 times to insert the device correctly. All the midwifes and physicians, with the exception of one, expressed a high degree of approval with regard to neonatal resuscitation by LMA and defined it a sustainable and cost-effective procedure. Table 3

## Discussion

Neonatal mortality, amounting to an estimated 4 million deaths worldwide each year takes place, in 98% of cases, in developing countries. As approximately 19% of these deaths are due to birth asphyxia [1], identifying solutions to achieve the Millennium Development Goal of halving child mortality by 2015 by means of a wide-scale implementation of cost-effective interventions has become urgent [17].

In this study, we proved the capability of learning and properly using in a manikin model LMA by physicians and midwifes, trained in a developing country to a NRP course and to a workshop on LMA. The knowledge gained by the physicians related to the LMA was superior than that achieved by the midwifes. Manikin-based comparative practical skills on LMA insertion showed instead, a similar high efficacy between trained physicians and midwifes, in terms of number of successful attempts and of time for placement. Unanimously, midwifes and physicians, except one, also manifested an high degree of approval of the neonatal resuscitation by LMA, giving a positive evaluation of his/her sustainability and cost-effectiveness in a low income country.

Some studies have demonstrated that NRP courses are effective in teaching neonatal resuscitation in developing countries [18] and have indicated approaches and techniques that have proved efficacious in preliminary trials in saving newborn lives [14,19]. Birth attendants in India [20] and China [21] involved in the NRP have been able to reduce asphyxia-related deaths. There is also evidence that mouth-to-mask and bag-and-mask resuscitation are comparable techniques with regards to neonatal mortality and morbidity [4,22]. The evidence that room air [23,24] and mouth-to-mask could be satisfactory used for neonatal resuscitation [25] suggests that firstly, it is reasonable to promote the basic elements of resuscitation of newborn resuscitation, as routine part of newborn care. Meanwhile, where feasible, all birth attendants should be trained to provide positive ventilation with other conventional methods currently used in neonatal resuscitation, bag-and-mask or bag-and-LMA ventilation.

The LMA may theoretically offer many practical (i.e. easier transport and sterilization), cost-effectiveness, and sustainable advantages (i.e. low cost coupled with the fact that it can be reused) over the face mask. As mentioned above, LMA allows a low pressure airtight seal against the glottis [7-9] and studies on the efficacy of ventilation by medical and paramedical personnel in neonatal training models have shown that the LMA combines ease of insertion and adequate, rapid airway patency [11].

The AHA recommends bag-and-mask ventilation, a challenging procedure for those inexperienced in neonatal resuscitation when a newborn requires PPV, while tracheal intubation may be impossible due to lack of skill or the presence of severe congenital abnormalities. Some case reports have, moreover, shown the successful use of the LMA in resuscitation of newborns with congenital airway abnormality under inadequate ventilation and difficult intubation settings [26,27]. Tests on neonatal intubation training models have shown that midwives and interns can obtain a clear airway more rapidly with LMA than TT and with fewer failures with LMA than with TT [28].

While our study demonstrates that LMA can be easily taught to local healthcare workers in developing countries, it does have some limitations. The theoretical knowledge gained and the manual skills involved in manipulating LMA and a manikin concern only a scant handful of birth attendants and were never verified in real asphyxiated neonates in the delivery room or in other birth settings. The cost-effectiveness and sustainability of resuscitation by LMA in countries where resources are extremely limited require further scientific scrutiny, just as it would be useful to analyze the retention of the knowledge and skills gained by these and other birth attendants attending specialized courses.

## Conclusions

In view of the minimum amount of time and resources necessary to train the participants in NRP courses and its many advantages with respect to FM and TT, LMA should be considered for further evaluation and use in developing countries.

## Competing interests

The authors declare that they have no competing interests.

## Authors' contributions

VZ had the original idea for strategy of analysis in collaboration with DT and AS. MM retrieved the data. LT reviewed the literature. FC did the statistical analysis. VZ wrote the paper. All authors read and approved the final manuscript.

## Pre-publication history

The pre-publication history for this paper can be accessed here:

http://www.biomedcentral.com/1472-6963/10/254/prepub

## Supplementary Material

Additional file 1 **Neonatal Resuscitation Program (NRP). Questions on neonatal Laryngeal Mask Airway**. Questions concerning: (I) features of LMA; (II) advantages of LMA over the face mask; (III) advantages of LMA over the endotracheal tube; (IV) disadvantages of LMA; and (V) potential applications in neonatal resuscitation. Click here for file

## References

[B1] Bhutta ZA Darmstadt GL Hasan BS Haws RA Community-based interventions for improving perinatal and neonatal health outcomes in developing countries: a review of the evidence Pediatrics 2005 115;519 617 15866863 10.1542/peds.2004-1441

[B2] The International Liaison Committee on Resuscitation (ILCOR) consensus on science with treatment recommendations for pediatric and neonatal patients: neonatal resuscitation Pediatrics 2006 117 e978 e988 10.1542/peds.2006-0350 16618791

[B3] Bloom RS Cropley C Textbook of Neonatal Resuscitation Elk Grove Village, IL. American Heart Association, American Accademy of Pediatrics 2000 4

[B4] Massawe A Kilewo C Irani S Verna RJ Chakrapam AB Ribbe T Assessment of mouth-to-mask ventilation in resuscitation of asphyxic newborn babies. A pilot study Trop Med Int Health 1996 1 865 873 10.1111/j.1365-3156.1996.tb00124.x 8980603

[B5] Trevisanuto D Ibrahim SA Doglioni N Salvadori S Ferrarese P Zanardo V Neonatal resuscitation courses for pediatric residents: comparison between Khartoum (Sudan) and Padova (Italy) Paediatr Anaesth 2007 17 28 31 10.1111/j.1460-9592.2006.02001.x 17184428

[B6] Grein AJ Weiner GM Laryngeal mask airway versus bag-mask ventilation or endotracheal intubation for neonatal resuscitation [protocol] Cochrane Library Document 2002 10.1002/14651858.CD003314.pub2 15846656

[B7] Brain AIJ Vergese C Addy EV The intubating laryngeal mask. I. Development of a new device for intubation of the trachea Br J Anaesth 1997 79 699 703 9496198 10.1093/bja/79.6.699

[B8] Brain AIJ Vergese C Addy EV Kapila A Brimacombe J The intubating laryngeal mask II: a preliminary clinical report of a new means of intubating the trachea Br J Anaesth 1997 79 704 709 9496199 10.1093/bja/79.6.704

[B9] Harnett M Kinirorns B Heffernan A Motherway C Casey W Airway complications in infants: comparison of laryngeal mask airway and the facemask-oral airway Can J Anaesth 2000 47 315 318 10.1007/BF03020944 10764174

[B10] Mora EU Weiner GM Alternative Ventilation Strategies: Laryngeal Masks Clin Perinatol 2006 33 99 110 10.1016/j.clp.2005.11.011 16533636

[B11] Micaglio M Doglioni N Parotto M Zanardo V Ori C Trevisanuto D Training for neonatal resuscitation with the laryngeal mask airway: a comparison of the LMA-ProSeal and the LMA-Classic in an airway management manikin Paediatr Anaesth 2006 16 1028 31 10.1111/j.1460-9592.2006.01921.x 16972830

[B12] Office of Population Research World fertility surveys 1973 Princeton, NJ: Office of Population Research, Princeton University http://opr.princeton.edu/archive/wfs Accessed December 28, 2004

[B13] Deorari AK Paul VK Singh M Vidyasagar D Impact of education and training on neonatal resuscitation practices in 14 teaching hospitals in India Ann Trop Paediatr 2001 21 29 33 10.1080/02724930020028885 11284243

[B14] Ergenekon E Koc E Atalay Y Soysal S Neonatal resuscitation course experience in Turkey Resuscitation 2000 45 225 227 10.1016/S0300-9572(00)00179-9 10959023

[B15] Trevisanuto D Ferrarese P Cavicchioli P Fasson A Zanardo V Zacchello F Knowledge gained by pediatric residents after neonatal resuscitation program courses Pediatr Anesth 2005 15 944 947 10.1111/j.1460-9592.2005.01589.x 16238554

[B16] Martin PD Cyna AM Hunter WAH Henry J Ramayya GP Training nursing staff in airway management for resuscitation: a clinical comparison of the face mask and laryngeal mask Anaesthesia 1993 18 33 37 8434745

[B17] Bhutta ZA Gupta I de'Silva H Manandhar D Awasthi S Hossain SM Salam MA Maternal and child health: is South Asia ready for change? BMJ 2004 328 816 819 10.1136/bmj.328.7443.816 15070640 PMC383381

[B18] Marsh D Darmstadt G Moore J Daly P Oot D Tinker A Advancing newborn health and survival in developing countries: a conceptual framework J Perinatol 2002 22 572 576 10.1038/sj.jp.7210793 12368975

[B19] Darmstadt G Lawn J Costello A Advancing the state of the world's newborns Bull World Health Organ 2003 81 224 225 12764520 PMC2572417

[B20] Deorari A Paul V Singh M Vidyasagar D The national movement of neonatal resuscitation in India J Trop Pediatr 2000 46 315 317 10.1093/tropej/46.5.315 11077947

[B21] Zhu X Fang H Zeng S Li Y Lin H Shi S The impact of the neonatal resuscitation program guidelines (NRPG) on the neonatal mortality in a hospital in Zhuhai, China Singapore Med J 1997 38 485 487 9550910

[B22] Kamenir S Neonatal resuscitation and newborn outcomes in rural Kenya J Trop Pediatr 1997 43 170 173 10.1093/tropej/43.3.170 9231639

[B23] Saugstad O Rootwelt T Aalen O Resuscitation of asphyxiated newborn infants with room air or oxygen: an international controlled trial: the Resair 2 study Pediatrics 1998 102 1 10.1542/peds.102.1.e1 9651453

[B24] Ramji S Ahuja S Thirupuram S Rootwelt T Rooth G Saugstad O Resuscitation of asphyxic newborn infants with room air or 100% oxygen Pediatr Res 1993 34 809 812 10.1203/00006450-199312000-00023 8108199

[B25] Kamenir S Neonatal resuscitation and newborn outcomes in rural Kenya J Trop Pediatr 1997 43 170 173 10.1093/tropej/43.3.170 9231639

[B26] Mawer RJ Equipment for pediatric resuscitation Anaesthesia 1995 50 1 87 88 10.1111/j.1365-2044.1995.tb04530.x 7702161

[B27] Baraka A Larynx mask airway for resuscitation of a newborn with Pierre-Robin syndrome Anesthesiology 1995 80 1248 1253 10.1097/00000542-199509000-00039 7661375

[B28] Lavies NG Use of the laryngeal mask airway in neonatal resuscitation Anaesthesia 1993 48 352 10.1111/j.1365-2044.1993.tb06981.x 8494154

