# Supplementary material for: Laryngeal Mask Airway for neonatal resuscitation in a developing country: evaluation of an educational intervention. Neonatal LMA: an educational intervention in DRC
Source: BMC Health Serv Res. 2010 Aug 31;10:254. doi: 10.1186/1472-6963-10-254 (PMC2940880; doi:10.1186/1472-6963-10-254)
Supplement: Additional file 1 — Neonatal Resuscitation Program (NRP). Questions on neonatal Laryngeal Mask Airway. Questions concerning: (I) features of LMA; (II) advantages of LMA over the face mask; (III) advantages of LMA over the endotracheal tube; (IV) disadvantages of LMA; and (V) potential applications in neonatal resuscitation. [file 1472-6963-10-254-S1.DOC]

Additional file 1.

| **Neonatal Laryngeal Mask Airway** |
| --- |
| **Features YES/NO** |
| **Range (neonatal)**  **Size (neonatal)**  **Lubrication (neonatal)**  **Tip flattening**  **Advancing**  **Location**  **Cuff inflation**  **Connection for PPV**  **Correct positioning (neonatal)** |
| **Advantages over face mask: YES/NO** |
| **Less skill requires**  **Easy placement**  **Better airtight seal**  **Improved Sat. O_2_**  **Function uninfluenced by anatomical factors**  **Less hand fatigue** |
| **Advantages over tracheal intubation YES/NO** |
| **Increased speed and ease of placement by**  **trained medical and non-medical personnel**  **Avoid laryngoscopy**  **Avoid tracheal oedema**  **Efficacy in upper airway malformations when**  **intubation and mask ventilation fail**  **Avoid neuromuscular blocking agents use** |
| **Disadvantages YES/NO** |
| **Gastric insufflation and aspiration**  **Inadequate alveolar ventilation**  **Impossibility of suctioning the airway**  **Impossibility of administering drug endotracheally** |
| **Potential appliations in neonatal resuscitation YES/NO** |
| **When face mask and endotracheal tube**  **resuscitation fall**  **In neonatal training models allows a patent**  **airway in a shorter time than endotracheal tube**  **Incidence of failure low with LMA**  **Ruinously** |
